# Experimental data of adsorption of Cr(III) from aqueous solution using a bentonite: Optimization by response surface methodology

**DOI:** 10.1016/j.dib.2019.105022

**Published:** 2019-12-19

**Authors:** Johnatan D. Castro-Castro, Nancy R. Sanabria-González, Gloria I. Giraldo-Gómez

**Affiliations:** a Department of Physic and Chemistry, Universidad Nacional de Colombia sede Manizales, Campus La Nubia, km 7 vía al Aeropuerto, AA 127 Manizales, Colombia; b Department of Chemical Engineering, Universidad Nacional de Colombia sede Manizales, Campus La Nubia, km 7 vía al Aeropuerto, AA 127 Manizales, Colombia

**Keywords:** Bentonite, Adsorption, Trivalent chromium, Cr(III), Optimization, RSM

## Abstract

Experimental data of adsorption of Cr(III) from aqueous solutions using a Colombian bentonite were acquired. The adsorbent material was characterized by XRF, XRD, and nitrogen physisorption. The effect dataset of pH, agitation speed, contact time and adsorbent amount on the removal of Cr(III) from an aqueous solution, using sodium bentonite was reported. A complete factorial design 3^2^ with two replicates was used to estimate the influence of the adsorbent amount (0.50, 0.75 and 1.00 g) and pH (2.0, 3.0 and 4.0) on Cr(III) removal. Experimental dataset was evaluated with Design Expert® software using the response surface methodology (RSM) in order to obtain the interaction between the processed variables and the response. The optimal conditions for Cr(III) removal from aqueous solution of 50 mg/l were as follows: pH of 3.5, and the bentonite amount equals 0.96 g, keeping constant the contact time at 60 min and stirring speed at 250 rpm. The equilibrium isotherms at 25, 30 and 35 °C were fitted by means of the Langmuir and Freundlich models, and the respective parameters of such models were obtained. The maximum adsorption capacity of sodium bentonite to Cr(III) removal was between 6.44 ± 0.11 and 6.79 ± 0.21 mg/g in the temperature range from 25 to 35 °C. According to the experimental data acquired, sodium bentonite is an effective adsorbent for the Cr(III) removal from aqueous solutions, with the advantage of being a natural, abundant and low-cost material.

**Table:** Specifications Table

Subject	Environmental Science
Specific subject area	Environmental Chemistry Adsorption is a technique for the removal of inorganic pollutants such as heavy metals from water and wastewater
Type of data	Table Image Figure
How data were acquired	Chemical analysis was accomplished by X-ray fluorescence using a Magix Pro Philips PW2440 instrument with Rh 4 kV tube and samples prepared as pearls. The XRD patterns of the samples of adsorbent (oriented mounts) were taken to a Rigaku Miniflex II diffractometer with Cu Kα radiation, in the 2θ range of 3–60°, with a scan speed of 0.02° in 2θ/step, and counting time/step of 0.6 s. Nitrogen adsorption-desorption isotherms, measured at 77 K, were obtained with a Micromeritics ASAP-2020. The samples were degassed at 90 °C for 3 h and 150 °C for 2 h in vacuum, prior to the measurements at 77 K. The chromium concentration was measured by atomic absorption spectrophotometry with the flame technique using a Thermo Scientific iCE 3000 Series spectrometer. All graphical were acquired using OriginPro 8.0® software (OriginLab Corporation, USA).
Data format	Raw Analyzed
Parameters for data collection	All experimental data of batch adsorption were manually recorded. Cr(III) removal was calculated from the initial and final concentrations of Cr(III). The adsorption isotherms were obtained at initial concentrations of Cr(III) in the range of 50–200 mg/l and at temperatures of 25, 30 and 35 °C, at the optimal conditions found in the experimental design: 0.96 g adsorbent amount and pH equals 3.5.
Description of data collection	The dataset of effects of experimental parameters (pH, stirring speed, contact time and adsorbent amount) in the Cr(III) removal on bentonite were acquired by batch adsorption tests. Experimental dataset of the complete factorial design 3^2^ was processed with Design-Expert software using the response surface methodology (RSM). Parameters of the Langmuir and Freundlich isotherms were calculated using nonlinear regression of the data with software OriginPro 8.0®. The supplementary material contains two Excel files with data of adsorbent characterization and adsorption experiments.
Data source location	Universidad Nacional de Colombia sede Manizales Manizales city Colombia 5° 01′ 45″ N, 75° 28′ 21″ W
Data accessibility	With the article

**Table:** 

**Value of Data** • The data are useful describe how pollutant interact with the adsorbent material (adsorption mechanism, surface properties and adsorption capacity) and thus are essential for design of the adsorption systems. • These data offer an efficient method for the removal of Cr(III) in aqueous solution by exchange cationic and can be used for industries that generate effluents with Cr(III) and others heavy metal cations. • With the data of optimal adsorption conditions of Cr(III) onto bentonite, can be developed experiments to establish the kinetic and thermodynamic parameters of process. • These data helps predict the effect of parameters governing the adsorption process, especially when the adsorbent is a montmorillonite-type clay. • The data showed that sodium bentonite is highly efficient for the Cr(III) removal from aqueous solution, with the advantage of being a natural, abundant and low-cost adsorbent material.

## 1 Data description

**Fig. 1:**
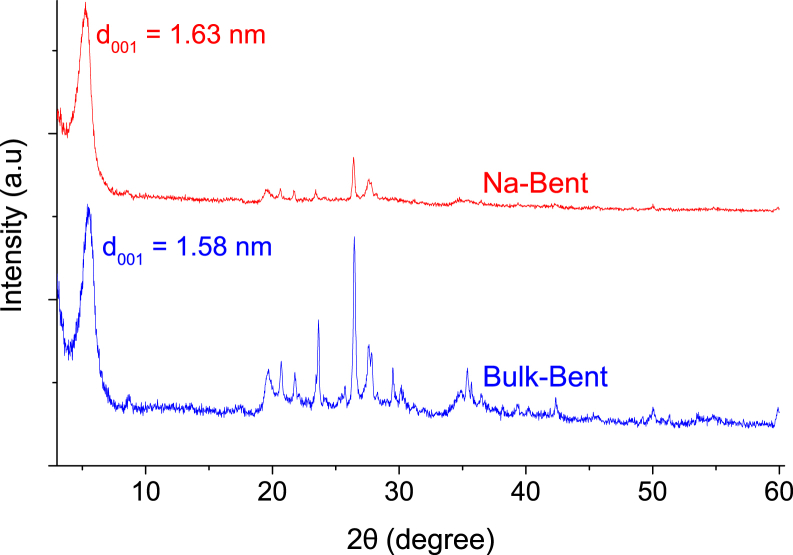
XRD patterns of bulk bentonite and of bentonite purified and subsequently exchanged with sodium.

**Fig. 2:**
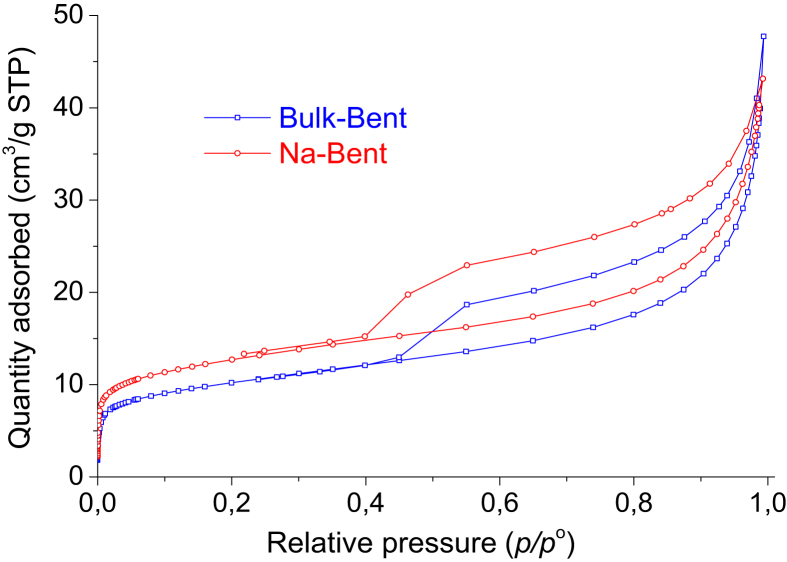
Nitrogen adsorption-desorption isotherms of bulk bentonite and bentonite purified and subsequently exchanged with sodium.

**Fig. 3:**
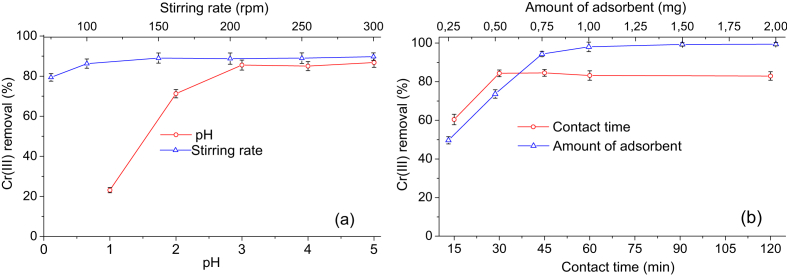
Cr(III) removal as a function of: a) pH and stirring speed, b) contact time and amount of adsorbent.

**Fig. 4:**
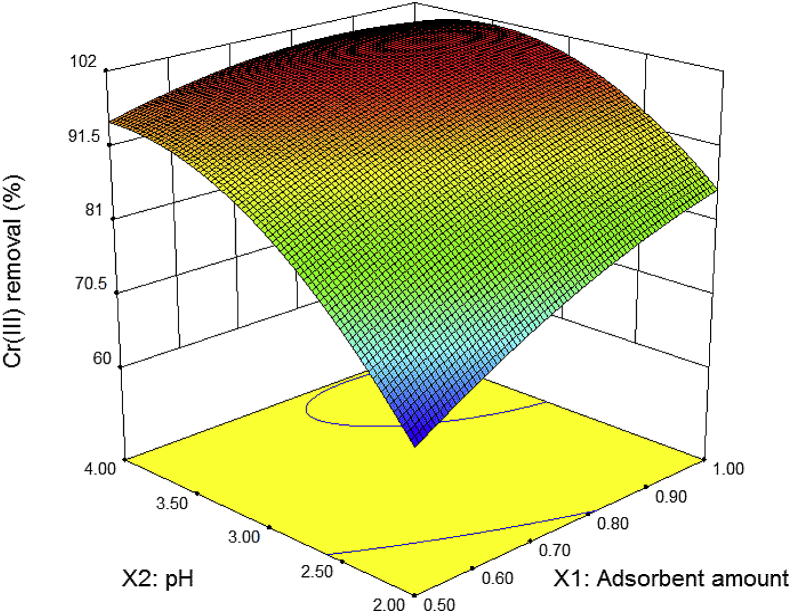
3D surface plot for Cr(III) removal as a function of pH and the amount of adsorbent.

**Fig. 5:**
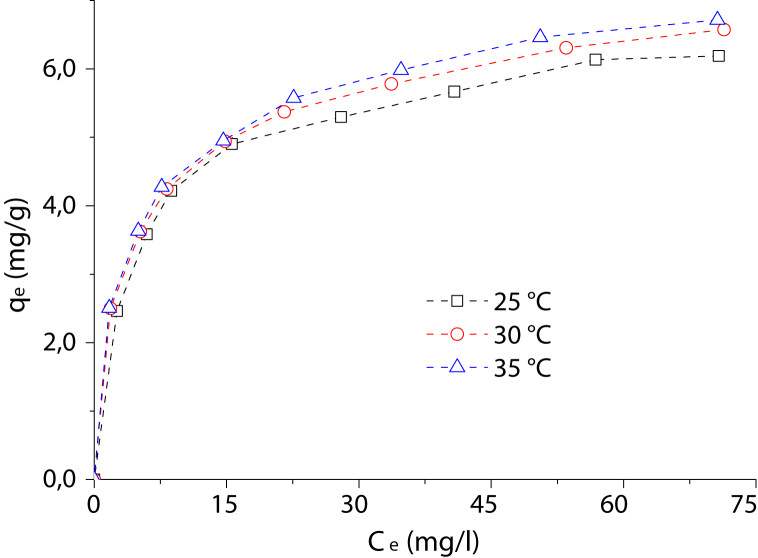
Adsorption isotherms of Cr(III) onto Na-Bent at different temperatures.

This dataset contains seven Tables and five Figures. The XRF analysis of bulk bentonite (so-called Bulk-Bent) and clay after purification, homoionized with sodium ions (so-called Na-Bent), are listed in Table 1. The XRD patterns of Bulk-Bent and Na-Bent are shown in Fig. 1. Nitrogen adsorption-desorption isotherms of Bulk-Bent and Na-Bent are illustrated in Fig. 2. Table 2 shows the experimental conditions used to evaluate the Cr(III) adsorption based on the pH, stirred speed, contact time and adsorbent mass. Cr(III) removal as a function of pH, stirring speed, contact time and amount of adsorbent are presented in Fig. 3(a) and (b). The experimental design data for the independent variables pH and amount adsorbent, with the recorded response, are shown in Table 3. ANOVA data for the second order model in the Cr(III) removal is shown in Table 4, and the 3D graphic for the design is illustrated in Fig. 4. Fig. 5 shows the data of the adsorption isotherms of Cr(III) onto Na-Bent at different temperatures. The equations of Langmuir and Freundlich describing the adsorption isotherms models are shown in Table 5. The equilibrium parameters estimated from the nonlinear fit of the data to the Langmuir and Freundlich models are offered in Table 6. The comparison between the adsorption capacity of Cr(III) and other adsorbents is shown in Table 7.

**Table 1:** Chemical analysis of bulk bentonite and bentonite purified and subsequently exchanged with sodium.

Compound	Bulk-Bent, (%)	Na-Bent, (%)
SiO_2_	57.81	55.04
Al_2_O_3_	16.25	15.67
Fe_2_O_3_	8.03	7.59
CaO	3.51	1.12
MgO	2.35	2.26
K_2_O	1.78	1.47
Na_2_O	1.31	2.12
TiO_2_	0.69	0.67
MnO	0.13	0.12

**Table 2:** Experimental conditions to evaluate the adsorption of Cr(III) independently analyzed the effect of each factor.

Test	pH	Stirring speed (rpm)	Contact time (min)	Adsorbent amount (g)
1	1.0, 2.0, 3.0, 4.0, 5.0	150	60	0.50
2	3	50, 100, 150, 200, 250, 300	60	0.50
3	3	150	15, 30, 45, 60, 120	0.50
4	3	150	60	0.25, 0.50, 0.75, 1.00, 1.50, 2.00

**Table 3:** Factorial design for the independent variables used along with the observed response.

Run	Amount of adsorbent (g)	pH	Cr(III) removal (%)
1	0.50	2.0	65.98 ± 0.36
2	0.75	2.0	77.96 ± 0.73
3	1.00	2.0	85.86 ± 0.12
4	0.50	3.0	87.76 ± 0.20
5	0.75	3.0	97.02 ± 0.19
6	1.00	3.0	99.19 ± 0.02
7	0.50	4.0	95.25 ± 0.77
8	0.75	4.0	98.79 ± 0.20
9	1.00	4.0	99.43 ± 0.07

**Table 4:** Analysis of variance (ANOVA) for second order model in the removal of Cr(III) on Na-Bent.

Source	Sum of square	GL	Mean square	*F-value*	*p*-value
Model	3206.62	5	641.32	1730.38	<0.0001
X1: Amount of adsorbent	155.78	1	155.78	420.32	<0.0001
X2: pH	1521.30	1	1521.30	4104.67	<0.0001
X1X2	184.82	1	184.82	498.68	<0.0001
X1X1	33.03	1	33.03	89.12	<0.0001
X2X2	332.31	1	332.31	896.63	<0.0001

**Table 5:** Adsorption isotherms models by Langmuir and Freundlich.

Model	Equation	Description	Ref
Langmuir	$q_{e}=\frac{Q_{0}K_{L}C_{e}}{1+K_{L}C_{e}}$ $R_{L}=\frac{1}{1+K_{L}C_{0}}$	$q_{e}$: amount of solute adsorbed in the adsorbent (mg/g) $Q_{0}$: maximum adsorption capacity (mg/g) $K_{L}$: constant according to the Langmuir model (l/mg) $R_{L}$: separation factor (dimensionless)	[4,5]
Freundlich	$q_{e}=K_{F}C_{e}^{\frac{1}{n}}$	$q_{e}$: amount of solute adsorbed in the adsorbent (mg/g) $K_{F}$: Freundlich constant related to the adsorption capacity ((mg/g)(l/mg)^n^) *n*: Freundlich constants related to the adsorption intensity of the adsorbent (dimensionless)	[4,6]

**Table 6:** Equilibrium isotherm parameters for the adsorption of Cr(III) onto Na-Bent.

Model	25 °C	30 °C	35 °C
**Langmuir**			
$Q_{0}$ (mg/g)	6.4373 ± 0.1070	6.6347 ± 0.1924	6.7900 ± 0.2117
$K_{L}$ (l/mg)	0.2163 ± 0.0165	0.2391 ± 0.0321	0.2417 ± 0.0349
$R_{L}$ (dimensionless)	[0.024–0.085]	[0.021–0.077]	[0.020–0.076]
$R^{2}$	0.9954	0.9866	0.9844
**Freundlich**			
$K_{F}$ ((mg/g)(l/g)^n^)	2.3794 ± 0.1796	2.4980 ± 0.1361	2.5230 ± 0.1324
n (dimensionless)	4.2699 ± 0.3973	4.2545 ± 0.2907	4.1747 ± 0.2697
$R^{2}$	0.9831	0.9902	0.9911

**Table 7:** Comparison of the maximum adsorption capacity of different low cost adsorbents for the adsorption of Cr(III).

Adsorbents	$Q_{0}$ (mg/g)	Ref
Natural sepiolite	0.53	[7]
Kaolinite	0.73	[8]
Bentonite	13.79	[9]
Bentonite GMZ	4.68	[10]
Diatomite	6.90	[11]
Na-Bent	6.44	This data article

## 2 Experimental design, materials, and methods

### 2.1 Materials

A stock solution of 1000 mg/l of Cr(III) was prepared from standard solutions of Titrisol Merck® (CrCl_3_ in HCl 4.2%); then it was used to make the solutions by means of appropriate dilution with double-distilled water. The pH of the solutions was adjusted by adding a 0.1 M HCl or NaOH solution. HCl (37% purity), NaOH (≥97 purity) and NaCl (≥99.5 purity) were purchased from Merck KGaA (Germany). The bulk clay used was a bentonite from Armero-Guayabal, municipality in the north of the department of Tolima - Colombia. This clay was supplied by Gea Minerales S.A.S., which estimated to have ore reserves of around 1.2 million tons [1].

### 2.2 Purification and characterization of the clay

The bulk clay was dried at 45 °C for 24 h, ground and sieved in 100 mesh (particle size smaller than 149.86 μm). The separation by particle size of the clay fraction (<2 μm) was made by gravitational sedimentation [2] and then, the purified bentonite, was converted to sodium bentonite (denoted as Na-Bent) by two exchanges with the 0.1 M NaCl solution. The Na-Bent obtained was repeatedly washed with distilled water until the leachate tested negative for chloride ions, dried at 60 °C and, finally, ground and sieved in a 100 mesh. The data of the physicochemical characterization of the bulk clay and the bentonite purified and subsequently exchanged with sodium (Na-Bent), corresponding to XRF, XRD and N_2_ physisorption are shown in Table 1 and Fig. 1, Fig. 2, respectively.

### 2.3 Batch adsorption experiments

Batch adsorption experiments were carried out at ambient conditions (20 °C and 78 kPa of atmospheric pressure). For this, a 50 ml solution of Cr(III) of 50 mg/l was put into a 100 ml Erlenmeyer flask with an added amount of Na-Bent. The mix was magnetically shaken at constant speed using an MS-H280-PRO hotplate stirrer (Germany) for a given time. At the end of the test time, the adsorbent was separated by filtration (0.45 μm millipore filter), and the concentration of chromium present in the aqueous solution measured by atomic absorption spectrometry (iCE 3500, Thermo Scientific, Germany) with an air-acetylene flame. The chromium removal was determined from the following equation:

(1) $$\text{Cr}\left(\text{III}\right)\text{ removal }\left(\text{\%}\right)=\;\frac{C_{o}-C_{t}}{C_{o}}\times 100$$

where $C_{o}$ is the initial concentration of Cr(VI) and $C_{t}$ is the concentration of Cr(III) on the time *t* (mg/l). All tests were performed in triplicate.

Experimental conditions to evaluated the adsorption of Cr(III) considered the effect of each factor (pH, agitation speed, adsorbent mass and contact time) keeping the other parameters constant, as indicated in Table 2. The pH of the solution was adjusted with 0.1 M HCl and NaOH solutions, and monitored with an SI Analytics Lab 845 pH meter (Germany). The Cr(III) removal as a function of pH and stirring speed are shown in Fig. 3(a), while the effect of contact time and the amount of adsorbent are found in Fig. 3(b).

### 2.4 Experimental design and adsorption isotherms

With the data of the initial tests performed on the experimental conditions shown in Table 2, a complete factorial design 3^2^ was established, having the amount of adsorbent (X1: 0.50, 0.75 and 1.00 g) and the pH (X2: 2.0, 3.0 and 4.0) as variables. All tests were performed in triplicate and keeping the following parameters constant: concentration of Cr(III) at 50 mg/l, contact time at 60 min, and agitation speed at 250 rpm.

The ranges of the factors were determined from the initial tests of the effects of the adsorption process variables. The Cr(III) removal was established as the response variable. Experimental data were evaluated with a Design Expert® software version 8.0 (StatEase, Inc., Minneapolis, MN, USA) using a response surface methodology (RSM). The interactions between independent factors were determined with analysis of variance (ANOVA) and the main effects of Cr(III) adsorption were identified based on the *p*-value with >95% of confidence level. Data were adjusted to a second-order polynomial equation to determine the coefficients of the response model as well as their standard errors and significance [3].

The design matrix with the values of the independent variables (amount of adsorbent and pH) used in the runs and the data of Cr(III) removal obtained are shown in Table 3. ANOVA data for Cr(III) removal on Na-Bent are shown in Table 4. The empirical model equation for Cr(VI) removal on Na-Bent can be described by the following equation:

(2) $$\text{Cr(III) removal (\%)}\;=\;-76.7744\;+\;127.0628\text{X}1\;+\;67.0380\text{X}2\;-\;{37.5404\text{X}1}^{2}\;-\;15.6982\text{X}1\text{X}2\;-\;{7.4422\text{X}2}^{2}$$

$${\text{R}}^{2}\;=\;0.9976\text{,}\;{\text{R}}^{2}\text{ adjusted}\;=\;0.9970$$

Fig. 4 shows a three-dimensional (3D) response surface plot as a function of adsorbent amount and pH. The adsorption isotherms of Cr(III) were realized at initial concentrations of Cr(III) in the range of 50–200 mg/l and at temperatures of 25, 30 and 35 °C, at the optimal conditions obtained in the experimental design: 0.96 g adsorbent and pH of 3.5. The adsorption capacity of Cr(III) so-called $q_{e}$ (mg/g), was quantified by the following expression:

(3) $$q_{e}=\frac{\left(C_{o}-C_{e}\right)\times V}{m}$$

where $q_{e}$ is the amount of solute adsorbed in the adsorbent (mg/g), $C_{o}$ and $C_{e}$ are the initial and equilibrium concentrations of Cr(III) in aqueous solution (mg/l), *V* is the solution volume (l), and *m* is the mass of the adsorbent (g). The adsorption isotherms of Cr(III) on Bent-Na at the three selected temperatures are shown in Fig. 5. Equilibrium isotherms data were fitted to Langmuir and Freundlich equations described in Table 5, and constants of isotherm equations were determined (Table 6). The maximum adsorption capacity of Cr(III) obtained onto Na-Bent was compared with that of other adsorbents in the literature, as shown in Table 7.
